# Contextual considerations for deception production and detection in forensic interviews

**DOI:** 10.3389/fpsyg.2023.1134052

**Published:** 2023-02-07

**Authors:** David M. Markowitz, Jeffrey T. Hancock, Michael T. Woodworth, Maxwell Ely

**Affiliations:** ^1^School of Journalism and Communication, University of Oregon, Eugene, OR, United States; ^2^Department of Communication, Stanford University, Stanford, CA, United States; ^3^Department of Psychology, University of British Columbia Okanagan, Kelowna, BC, Canada

**Keywords:** deception, lying, context, cold model, language, forensic interviewing

## Abstract

Most deception scholars agree that deception production and deception detection effects often display mixed results across settings. For example, some liars use more emotion than truth-tellers when discussing fake opinions on abortion, but not when communicating fake distress. Similarly, verbal and nonverbal cues are often inconsistent predictors to assist in deception detection, leading to mixed accuracies and detection rates. Why are lie production and detection effects typically inconsistent? In this piece, we argue that aspects of the context are often unconsidered in how lies are produced and detected. Greater theory-building related to contextual constraints of deception are therefore required. We reintroduce and extend the Contextual Organization of Language and Deception (COLD) model, a framework that outlines how psychological dynamics, pragmatic goals, and genre conventions are aspects of the context that moderate the relationship between deception and communication behavior such as language. We extend this foundation by proposing three additional aspects of the context — individual differences, situational opportunities for deception, and interpersonal characteristics — for the COLD model that can specifically inform and potentially improve forensic interviewing. We conclude with a forward-looking perspective for deception researchers and practitioners related to the need for more theoretical explication of deception and its detection related to the context.

## Introduction

Deception production and detection are contingent phenomena. How people tell lies, what they lie about, and how well people can detect lies can vary across settings or depend on a range of factors. For example, the lies people tell about potentially controversial opinions (Newman et al., 2003) are different than the lies people tell during a fake 9-1-1 emergency call in terms of their emotional content (Burns and Moffitt, 2014). People who lie about their abortion views tend to overuse negative affect compared to truth-tellers, and people who lied about medical emergencies underuse negative affect compared to truth-tellers. The personality constructs of psychopathy and Machiavellianism have not only been associated with the propensity to lie, but also the amount of positive emotion experienced in deceiving others (Baughman et al., 2014). Lie detection accuracy is also dependent on artifacts of truth-lie judgments (e.g., lie-truth base-rates; Levine et al., 1999). Accuracy for truths is often greater than accuracy for lies because people tend to guess “true” more often than “false” in detection tasks (Levine, 2014, 2020). Together, across decades of empirical scholarship and hundreds of studies, one of the most stable findings in deception research is that telling lies and detecting lies are impacted by *the context*. However, theoretical conceptualizations of the context for deception research are uncommon (for exceptions, see Blair et al., 2010; Markowitz and Hancock, 2019), despite many papers and empirical investigations calling for a better understanding of what it means. The context is often a catch-all to describe why lie production or detection may differ across settings. This universal application of the context leads to conceptual opacity instead of clarity, which we hope to alleviate in this paper.

Here, we draw on and expand existing theoretical models to explicate aspects of the context that matter for deception production and its detection. We specifically focus our efforts on identifying how aspects of the context inform our understanding of deception production and detection in forensic interviewing, which will impact empirical research and practice to diagnose lies from truths. Existing scholarship has articulated how contextual characteristics (e.g., pragmatic goals) inform the relationship between deception and language (Markowitz and Hancock, 2019), though our aims are broader, as we attempt to build on this foundation by considering more contextual factors that modify how people lie and detect lies.

## Deception and context: A current overview of the literature

Interest in the role of the context for deception research appears to be mixed over time. To validate this claim, we extracted academic abstracts from January 1, 2012 to October 2022 that contained the terms *deception* or *lying* as subjects using the first author’s university library system. This resulted in a corpus of over 16,000 peer-reviewed papers over the decade. This number was reduced to 9,614 after removing duplicates and irrelevant pieces. We identified the number of articles that mentioned at least one term related to the context (e.g., *context, contextual*, *contextually* etc.), and chose abstracts because they are succinct summaries of the research. If the words *context* or *contextual* appeared in such short texts, this would indicate that they were a key focus for the authors, as opposed to being an afterthought in the Discussion.

**Figure 1:**
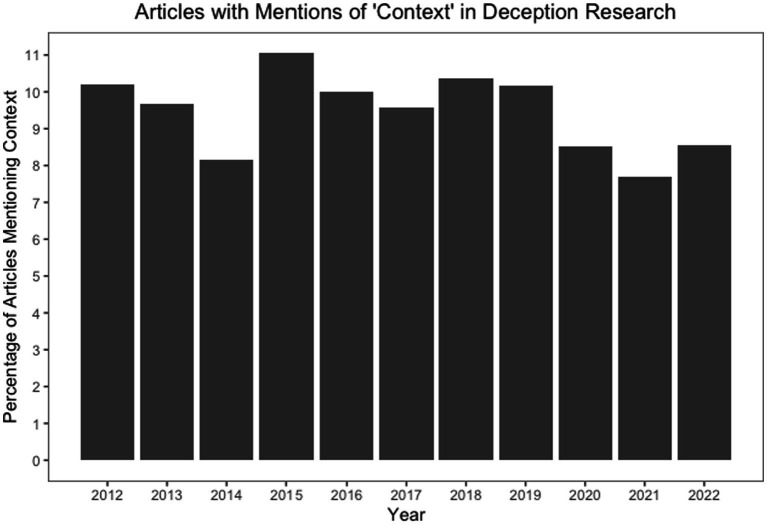
Frequency of context-related terms in the deception literature over time. The total number of articles in this analysis was 9,614.

A total of 901 papers focused on context (9.4%),^1^ and the data in Figure 1 suggest the frequency of context-focused papers vacillates. This evidence is illustrative because it suggests a nontrivial number of deception papers attend to aspects of the context in the research process and in academic reports, though the majority do not. To understand why there are mixed effects across deception studies and how to perhaps resolve them, a greater understanding and treatment of the context is needed in the published literature.

Having established that deception scholarship has a limited focus on the context in the past decade, it is now important to consider how scholars have thought about the role of the context in terms of empirical evidence and theory. Perhaps the most compelling evidence suggesting that contextual factors impact deception production originates from a meta-analysis by Hauch et al. (2015). The authors observed that in over 40 studies, the relationship between deception and language was systematic for many verbal dimensions (e.g., emotion, cognitive complexity), though the effect sizes were small. Crucially, five moderators often changed the nature of the relationship between deception and language (e.g., the event type, the emotional valence of the situation, the intensity of the interaction, motivation, and the production mode). For example, as others have noted (Markowitz and Hancock, 2019, 2022), liars typically use fewer words than truth tellers in their verbal accounts. This effect, however, is moderated by the interaction level between communicators (e.g., no interaction, computer-mediated communication, an interview, or a person-to-person interaction): liars tend to use more words than truth-tellers online, but fewer words without an interaction, when the interaction is an interview, or when it is person-to-person. The Hauch et al. (2015) meta-analysis provides clear evidence that the context and contextual factors matter for deception, yet our conceptualizations of the context are limited.

One theoretical model, however — the Contextual Organization of Language and Deception (COLD) framework — prescribes how the context may impact the relationship between deception and communication behavior such as language. Specifically, there are three aspects of the context that matters for the relationship between deception and language: (1) psychological dynamics, (2) pragmatic goals, and (3) genre conventions. Psychological dynamics relate to the emotional and cognitive experiences of a liar, which may be different than a truth-teller, and are often inconsistent across deceptions. Comparing primary study effects for the same linguistic indicator can help to demonstrate the impact of psychological dynamics on language patterns for deception. Prior online dating research observed that those who had more inaccurate items in their profile tended to focus on less negative emotion in the “about me” section of their dating advertisement compared to those who had more accurate items in their profile (Toma and Hancock, 2012). In a different deception setting, those who wrote false opinions related to abortion or their friends tended to focus on more negative emotion compared to those who wrote truthful opinions (Newman et al., 2003; Markowitz and Griffin, 2020). Using just these two examples, the same indicator (e.g., negative emotion terms) had a different relationship to deception that was modified by how people were thinking and feeling, or what they were attending to, at the time of lying or truth-telling.

Whether a person is apt to engage in deception at all can be tied to psychological dynamics of the situation as well. Mercadante and Tracy (2022) found that individuals who were labeled as “hubristically proud” (e.g., associated with low self-esteem, arrogance, and antisocial traits) were only more prone to lie when their status was threatened, and not in situations that were non-social or perceived as less threatening. Together, psychological aspects of a deception are critical contextual factors that change how people communicate about lies, an idea supported by decades of deception research across multiple domains, lie types, stakes, and settings (Ekman, 1989, 2001; Frank and Ekman, 1997).^2^

A second aspect of the context for deception and language relates to pragmatic goals. What people are trying to accomplish with their deception often changes how they falsely or truthfully communicate. Markowitz and Hancock (2019), in their evaluation of presidential lies, observed that those who lied about a rationale for war (e.g., President George W. Bush and President Lyndon B. Johnson) had a different linguistic profile and focus than those who lied because of a personal embarrassment (e.g., President Bill Clinton and President Richard Nixon). That is, the self-focus of these presidents was modified by what they were trying to accomplish. Presidents who were trying to convince the country of a contested war effort experienced a psychological distancing effect (e.g., a reduction in “I”-words in their lies compared to truths), whereas presidents who were trying to maintain their credibility after a personal and public humiliation experienced a psychological immediacy effect (e.g., an increase in “I”-words in their lies compared to truths; Weiner and Mehrabian, 1968). Goals are critical in deception research (Buller and Burgoon, 1996; Bond et al., 2013; Levine et al., 2016), and no two deceptions (nor deceivers) may have the same reasons for lying. Motivational attitudes and values also help facilitate our understanding of when an individual may be most inclined to dishonesty. For example, Lee et al. (2020) found that neutralization (e.g., overriding a social norm and justifying immorality) was the primary motivator for academic dishonesty. This evidence suggests a deeper consideration of how goals/motives modify communication and deceptive patterns is required.

A final aspect of the COLD model, genre conventions, draws on linguistics research to suggest how people communicate within “discourse communities,” which have norms that shape behavior (Biber et al., 2007). Independent of deception, discourse communities suggest ways of communicating either implicitly or explicitly. For example, an implicit discourse community norm includes the idea of not swearing in a religious building, while an explicit discourse community norm includes rules to ban hate speech on certain online forums (Twitter, 2022). A critical function of genre conventions is to identify what is normative and non-normative for people to communicate within a particular setting. Discourse communities change and shift within social interactions even prior to deception being communicated. Baseline communication conventions are important to acknowledge as a contextual factor that can also modify how people lie or tell the truth across deceptions (Markowitz and Griffin, 2020). Altogether, the COLD model is largely a lie production framework that attempts to articulate various contextual factors that impact how people communicate verbally when they lie versus tell the truth. Since its creation, however, we — the original authors of the COLD model and other collaborators — have considered other context-related factors that are also likely to impact lie production and detection. Our aim with the remainder of this piece is to outline new directions for the COLD model and apply them to forensic interviewing, focusing on how the model can be extended with new lie production characteristics (which can have implications for detection as well). We use existing empirical evidence to ground our additions to the COLD model and encourage other scholars to continue adding to this non-exhaustive foundation.

## Individual-level factors: Demographics and personality traits

Individual differences, including demographics and personality traits, have historically received limited treatment in the deception literature. While prior work has indeed suggested the role that certain dispositional traits may play in deceptive communication (e.g., self-monitoring; Miller and Stiff, 1993), recent work has offered even greater attention on individual-level characteristics to identify deception in a range of settings. General inclinations toward honesty can be identified using personality models such as the HEXACO model (Ashton et al., 2014), with evidence suggesting people who are high on honesty—humility tend to cheat less than people who are low on honesty—humility, on average (Markowitz and Levine, 2021). Personality traits beyond The Big Five and its derivations (John and Srivastava, 1999) have also identified people who are inclined to lie, cheat, or deceive in different deception settings. For example, the Dark Triad (Furnham et al., 2013; Jones and Paulhus, 2014) consists of three aversive personality traits: narcissism (e.g., people who are entitled and believe they are superior), Machiavellianism (e.g., people who are manipulative), and psychopathy (e.g., people who are generally less empathic and less anxious about misdeeds). What links these problematic personality traits is arguably manipulation and deceptive intent, with a recent study demonstrating that manipulativeness and dishonesty were some of the key characteristics defining psychopathy (Crego and Widiger, 2022). People who are high on such aversive personality traits tend to display more cheating behavior in some settings (Jones and Paulhus, 2017), and report higher-than-average lying self-reported lying rates than those who are low on such aversive personality traits (Daiku et al., 2021; Markowitz, 2022).

Deception studies may control for individual differences, but they might serve as key moderators for lie production or detection. Some people may be more dispositionally honest or deceptive than others (Jones and Paulhus, 2017; Markowitz and Levine, 2021), changing how often they lie and what they tend to lie about. We therefore suggest a natural extension of the COLD model is a focus on the individual and how certain underlying characteristics (e.g., personality traits) reveal deception across settings For example, Porter and Woodworth (2007) found that compared to non-psychopathic murderers, those scoring high on psychopathy were more likely to frame their offense in a reactive manner, downplaying the instrumental nature of the offense, and omitting specific details during an interview compared to those scoring low on psychopathy. A line of research has also found that individuals scoring high on psychopathy demonstrate unique language profiles indicative of low anxiety, less empathy, hostile and negative affect, as well as instrumental intentions compared to those scoring low on psychopathy (Hancock et al., 2013, 2018; Le et al., 2017). This less authentic and problematic language may not hinder their deceptive goals face-to-face (e.g., where they can also utilize nonverbal behavior), but in online environments, evidence suggests such people may have a reduced ability to manipulate others (Crossley et al., 2016).

Other types of individual differences (e.g., demographics) need greater treatment in the deception literature as well, since some work suggests they impact detection. One study evaluated how White students judged the veracity of Black and White targets, with evidence suggesting a greater truth-bias with Black compared to White targets (Lloyd et al., 2017). The effect was strongly related to one’s need to not appear prejudiced. However, in an eye-tracking study from the same paper, participants focused more on the word “lie” when the target was Black compared to White. These data have clear intergroup conflict and intergroup dynamic implications (Giles, 2012; Dunbar, 2017), but they also motivate a greater need to use demographics as a contextual and moderating factor in deception detection research. The demographic makeup of the communicator and target of a deception matter.

## Situation-level factors: Lie prevalence and base-rates

During interpersonal deception, most people lie when honesty is a problem (Levine, 2020), or when the opportunity for deception is available and facilitates some form of significant personal gain. Most people are not egregious liars; they tend to lie *just a little bit* to still be perceived as a good person while getting ahead of others by lying (e.g., the fudge factor; Ariely, 2012). However, a small segment of the population engages in prolific lying, defined as greater-than-average lying during a one-time task. Prolific lying, with skewed prevalence distributions where most people are honest and a few people have above-average lying rates, has been established in US settings (Serota et al., 2010; Levine et al., 2013; Markowitz and Hancock, 2018; Markowitz, 2022), Japan (Daiku et al., 2021), South Korea (Park et al., 2021), and other locations. Therefore, a critical contextual and situational moderator of deception is lie-truth base-rates. As others suggest (Levine, 2014, 2020; Markowitz, 2020), base-rates indicate how often deception is prevalent and how often one should expect deception in a particular setting. In a setting with very little deception and an overwhelming amount of honesty (e.g., disinformation online), detection accuracy will be near 100% as predicted by Truth-Default Theory (Levine, 2014, 2020). Therefore, to detect lies effectively, researchers and practitioners should attempt to establish base-rates of deception that can signal the probability of lying in each setting. If deception is improbable or implausible (Walczyk et al., 2014), detection efforts may be futile. Detection efforts with a more evenly distributed lie-truth base-rate may be more effective.

It is important to note that prolific lying and identifying prolific liars are not the same empirical task. Prolific lying considers deception tendencies during a single opportunity for gain (Serota et al., 2010; Levine et al., 2013; Daiku et al., 2021; Markowitz, 2022). Prolific liars are individuals who demonstrate a repeated proclivity for taking up the opportunity for deception (Serota et al., 2021). Therefore, the number of repeated deceptions over a particular timespan may be informative for deception research as a moderator to enhance detection abilities. A prolific liar may leave behind more behavioral traces of their deception than a person who engages in prolific lying because there are more datapoints on their behavior. This, in turn, may increase deception detection ability. However, it is unclear if prolific liars are also more clever deceivers who may cover their tracks and avoid detection better than people who engage in opportunistic, prolific lying. This open question offers a program of research for future deception scholarship. It is also worth noting that prolific lying may be considered an individual difference as well and therefore, there is some level of overlap between the current and prior sections. For example, pathological and prolific lying, along with manipulativeness, are considered two of the 20 key characteristics of psychopathy.

## Interpersonal factors: Deception consensus and style matching

Deception production and detection research often focuses on the individual, specifically how they communicate lies and the degree to which people can detect such lies. Lies are communicated to a target, however, and the relationship between the liar and receiver requires greater treatment in the literature. Interpersonal Deception Theory (IDT) offers critical insights into interpersonal dynamics, specifically interactivity and motives, that can help to understand who people lie to and how detection efforts can be improved (Buller and Burgoon, 1996). However, IDT is largely an interpersonal theory from a face-to-face perspective. It is important to consider how other interpersonal communication characteristics, which might originate from online sources, can inform IDT and feed into the COLD model.

First, prior work suggest interpersonal perceptions of dishonesty are correlated in online (Markowitz and Hancock, 2018) and offline settings (Markowitz, 2022). For example, Markowitz and Hancock (2018) coined the *deception consensus effect*, an idea that suggests one’s lying rate is positively correlated with their perceptions of dishonesty for a given setting. Online daters who lied a lot tended to think that other daters were also lying a lot as well. Therefore, a critical moderator of deception production frequency might be one’s perspective on the how often deception occurs for a given setting (e.g., Markowitz, 2020). If a person believes that others in a community are lying at high rates, this may license them to lie at high rates as well. Expectations for deception also have important implications for deception detection. If detectors believe that social media has widespread deception, they may be more likely to guess that a message is false compared to true (e.g., a deception bias instead of truth bias; Luo et al., 2022). Further, if law enforcement believes that certain individuals are more likely to lie than others, they might use shoddy interrogation tactics or unjustly accuse suspects. Interpersonal and intergroup perceptions are essential in everyday communication (Abeyta and Giles, 2017), suggesting they should also be considered when deception is involved as well (Dunbar, 2017).

While perceptions of suspects during the forensic interview are important to understand how detectors might judge certain groups of people, such investigations require the active recruitment of one’s thoughts and feelings about a target. For example, to identify how someone feels about another person and if they are lying about these feelings, self-report data may be required to identify discrepancies between what people say and what they report *via* survey data. An alternative, but complementary approach might use language patterns to identify liking and affinity toward another person or group. A variety of studies have demonstrated the more that people match on their use of style words (e.g., articles, prepositions, pronouns), the more that two people tend to have more favorable interpersonal perceptions, cohesion, interest, liking, and better interactions (Gonzales et al., 2010; Ireland et al., 2011). This insight — using style words as markers of interpersonal interest and liking — can help forensic interviewers who want to understand the degree to which suspects feel psychologically connected to the interviewer as revealed at the language level. It is unclear if deceivers style match more in order to be psychologically closer to interviewers and closely monitor how they are perceived (DePaulo, 1992), or if they style match less to distance themselves from their target (Newman et al., 2003; ten Brinke and Porter, 2012; Markowitz and Griffin, 2020). This open question should be a prime candidate for future research. Importantly, style matching is closely associated with demonstrating empathy and building rapport, which may enable interviewers in forensic settings to obtain more accurate and relevant information from guarded suspects. Brimbal et al. (2021) found that for more guarded suspects who were being interviewed, rapport not only reduced their level of resistance (and increased cooperation), but also facilitated additional information retrieval and the chance to increase the amount of *accurate* information obtained compared to less guarded suspects.

## Conclusion and future directions

Few theoretical perspectives are equipped to address how the context moderates lie production and lie detection effects. In this work, we broadly reintroduce the COLD model and articulate new directions for contextual constraints that impact how deception is communicated and detected. We suggest that researchers can take this evidence and build it into their research designs to test how individual differences, lie-truth base-rates and situational opportunities for deception, and interpersonal dynamics can modify lying and lie detection. More experimental research is needed to assess how these constraints compare or moderate deception and language effects relative to others that might exist in the literature (Vrij, 2018, 2019; Nahari et al., 2019). The COLD model is also limited in that it cannot yet make predictions. Future iterations of the model can work toward becoming a deception theory by making “formal, testable, falsifiable propositions” to be used in future scholarship (McCornack et al., 2014, 351). Practitioners should assess how their current approaches to lie detection fare, and perhaps draw on the COLD model to identify warning signs of lie production across settings where context effects loom large For instance, interviewing models (such as the Phased Interview Model) that incorporate substantial rapport building strategies show promise in obtaining additional credible and investigation relevant information in serious crime investigations (Cooper et al., 2021). Forensic interviewing can benefit from a more systematic acknowledgment that contextual elements impact lie detection, and the COLD model offers many pathways to understand the possible constraints that the context places on lie detection efforts.

We aimed to provide a non-exhaustive list of potential additions to the COLD model based on recent research, though there may be others that warrant consideration. We are excited to work collaboratively with research teams and further develop theory related to deception and context, particularly around how people communicate lies and truths. Practitioners play a critical role in this process, as their “boots on the ground” knowledge can highlight researcher blind-spots about how lies are told outside of the laboratory. Interestingly, clinicians in therapeutic settings are voicing concerns of using videoconferencing (particularly in forensic settings such as determining competency to stand trial), with 79.7% worrying about the reliability and validity of their work compared to in person interactions (Trupp et al., 2021). Within more general clinical settings, it is also incredibly important for additional research to consider therapists’ ability to gauge client credibility across communication media, given the high stakes nature of some of the information provided (for example, level of suicidal intent). We encourage more cross-pollination between research and practice, as they are symbiotic for the study of deception.

Taken together, the evidence in deception research is often mixed and contingent across studies. We argue that incremental theory-building related to the context is essential to understand how people tell lies across deceptions and how detection accuracy might vary across deceptions. Our extension of the COLD model is another attempt to progressively build our theoretical basis of knowledge into how aspects of the context — psychological dynamics, pragmatic goals, genre conventions, and now individual differences, lie-truth base-rates and situational opportunities for deception, and interpersonal characteristics — moderate lie production and lie detection effects.

## Author contributions

DM wrote the manuscript. JH, MW, and ME provided critical feedback. ME collected the article abstracts and DM performed the analysis. All authors contributed to the article and approved the submitted version.

## Conflict of interest

The authors declare that the research was conducted in the absence of any commercial or financial relationships that could be construed as a potential conflict of interest.

## Publisher’s note

All claims expressed in this article are solely those of the authors and do not necessarily represent those of their affiliated organizations, or those of the publisher, the editors and the reviewers. Any product that may be evaluated in this article, or claim that may be made by its manufacturer, is not guaranteed or endorsed by the publisher.

## Footnotes

^1^Including the search terms *situation* and *situational* increased this percentage to 13.6% (1,304/9,614). This evidence suggests context-related terms are more common than situation-related terms, though the general focus on the context or situation remains relatively low. A similar trend in Figure 1 emerged for using situation terms, only.

^2^While we propose and present evidence suggesting psychological dynamics moderate the relationship between deception and language, there is mixed evidence for this claim as well (Hartwig and Bond, 2011). We acknowledge this evidence in the spirit of transparency and to encourage scholarship that identifies the boundary conditions of such effects.
